# microRNA-338-3p inhibits proliferation, migration, invasion, and EMT in osteosarcoma cells by targeting activator of 90 kDa heat shock protein ATPase homolog 1

**DOI:** 10.1186/s12935-018-0551-x

**Published:** 2018-04-02

**Authors:** Riliang Cao, Jianli Shao, Yabin Hu, Liang Wang, Zhizhong Li, Guodong Sun, Xiaoliang Gao

**Affiliations:** 1 Department of Pediatric Surgery, Guangdong Women and Children Hospital, Guangzhou, 511400 China; 2 Department of Orthopedic and Traumatology, First Affiliated Hospital, Jinan University, Guangzhou, 510632 China; 3 Department of Spinal Surgery, The Sixth Affiliated Hospital of Xinjiang Medical University, Ürümqi, 830002 Xinjiang China; 4 Department of Oncology, First Affiliated Hospital, Jinan University, Guangzhou, 510632 China

**Keywords:** Osteosarcoma, microRNA-338-3p, Activator of 90 kDa heat shock protein ATPase homolog 1, Tumor suppressor, Translational repression

## Abstract

**Background:**

Osteosarcoma (OS) is a rare, malignant bone tumor that primarily affects adolescents and has a high degree of malignancy and high incidence of recurrence and metastasis. Our study aimed to explore the role of miR-338-3p in OS cells.

**Methods:**

qRT-qPCR was performed to quantify miR-338-3p expression levels in OS tissue samples and in three common OS cell lines. MG-63 and Saos2 cells were separately transfected with miR-338-3p or NC mimics and miR-338-3p expression levels was determined by qRT-PCR. Cell proliferation was monitored using the Cell Counting Kit-8. Flow cytometer analysis was carried out to determine the distribution of cell cycle stages and apoptosis. Transwell assay was performed to measure the migratory and invasive capacities of MG-63 and Saos2 cells. The expression of Vimentin and E-cadherin was detected by western blot. Luciferase reporter assay, qRT-PCR and western blotting were performed to confirm the target of miR-338-3p.

**Results:**

Analysis by qRT-PCR revealed that miR-338-3p was downregulated in the tissue samples of 20 OS patients when compared with that in their matched adjacent non-tumor tissues. Furthermore, miR-338-3p was significantly downregulated in three common OS cell lines, namely, MG-63, Saos2, and HOS, when compared with that in the human osteoblast cell line hFOB1.19. Analysis by luciferase reporter assay, qRT-PCR, and western blotting revealed that activator of 90 kDa heat shock protein ATPase homolog 1 (AHSA1) is a direct target of miR-338-3p. miR-338-3p overexpression led to significant reduction in AHSA1 protein levels in MG63 and Saos2 cells. miR-338-3p overexpression reduced cell viability and migration and invasion behavior of MG63 and Saos2 cells. In addition, miR-338-3p overexpression suppressed epithelial–mesenchymal transition (EMT), induced a significant G1-phase arrest and did not affect the apoptosis in both MG-63 and Saos2 cells. Moreover, overexpression of AHSA1 reversed the inhibitory effect of miR-338-3p overexpression on proliferation, cell cycle, apoptosis, EMT, migration, and invasion of MG63 and Saos2 cells, thereby suggesting that miR-338-3p acts as a tumor suppressor in OS cells by targeting AHSA1.

**Conclusions:**

miR-338-3p/AHSA1 can serve as a potential therapeutic target for OS therapy.

## Background

Osteosarcoma (OS) is one of the most common primary bone malignancies that primarily affect adolescents, especially individuals aged 15–19 [1, 2]. OS has high degree of malignancy and high incidence of recurrence and metastasis. Although major advances in OS treatment have been achieved in the past several decade, such as chemotherapy and radiotherapy in the past several decades, prognosis for OS patients still remains poor [3]. Therefore, elucidating the molecular mechanisms underlying OS will contribute to the development of effective strategies for OS treatment and prognosis.

The fundamental molecular mechanisms underlying the development of OS remain unclear. However, oncogene or tumor suppressor gene-regulation disorders can trigger consistent cell proliferation, migration and invasion, and thereby accelerate OS development [4]. Activator of 90 kDa heat shock protein ATPase homolog 1 (AHSA1) is a chaperone of HSP90, which is involved in the maturation, stabilization/degradation, and function of oncogenic proteins [5]. Our previous study showed that AHSA1 has a higher expression profile in OS cells and knock-down of ASHA1 could suppress cell growth, migration and invasion, revealing the oncogenic role of ASHA1 in OS [6]. However, the regulation mechanism on the higher expression profile of ASHA1 in OS cells is not clear.

MicroRNAs (miRNAs) are single-stranded RNAs with lengths ranging from 21 to 23 nucleotides [7]. miRNAs downregulate the expression of target genes by inducing messenger RNA (mRNA) degradation or inhibiting the translation of target genes through imperfect base-pairing with their 3′-untranslated regions (3′UTRs) [8]. In many cancer cells, miRNAs play important roles in regulating cell proliferation, apoptosis, migration, invasion, angiopoiesis, and epithelial mesenchymal transformation [9–11]. miR-338-3p deregulation has been demonstrated to be involved in several types of human malignances. For example, miR-338-3p was found to inhibit growth, metastasis, and invasion of non-small cell lung cancer (NSCLC) cells [12, 13]. Further, in gastric cancer cells, miR-338-3p suppresses the epithelial–mesenchymal transition, proliferation, and migration [14, 15]. The abovementioned results indicate that miR-338-3p acts as a tumor suppressor gene in cancer cells. However, the role of miR-338-3p in OS cells remains unclear. In addition, a miR-338-3p-binding site was found in the 3′UTR of AHSA1. So we aimed to identify the association between miR-338-3p and AHSA1 in the present study.

Our results showed that miR-338-3p is downregulated in OS tissues and cell lines. miR-338-3p overexpression inhibited viability, epithelial–mesenchymal transition (EMT), migration, and invasion in MG63 and Saos2 cells. Furthermore, AHSA1 was identified as a direct target of miR-338-3p. AHSA1 overexpression reversed the miR-338-3p overexpression-induced suppression of proliferation, EMT, migration, and invasion of MG63 and Saos2 cells. All our results suggest that miR-338-3p acts as a tumor suppressor in OS cells by targeting AHSA1.

## Methods

### Clinical samples

Surgically resected paired OS and normal adjacent tissues (NAT) were obtained from patients who underwent radical resection at the First Affiliated Hospital, Jinan University (Guangzhou, P. R. China) from 2013 to 2015. Surgically removed tissues were quickly frozen in liquid nitrogen until analysis. All protocols involving the use of patient samples in this study were approved by the Medical Ethics Committee of the First Affiliated Hospital, Jinan University (Guangzhou, P. R. China). A signed informed consent was obtained from each patient.

### Cell culture

Human OS cell lines MG-63, Saos2, and HOS, and the conditionally immortalized human fetal osteoblastic cell line hFOB1.19 were purchased from the Institute of Cell Bank/Institutes for Biological Sciences (Shanghai, China). MG-63, Saos2, and HOS cells were cultured in Dulbecco’s modified Eagle’s medium (DMEM) supplemented with 10% fetal bovine serum (FBS) in a humidified incubator with 95% air and 5% CO_2_ at 37 °C. hFOB1.19 cells were maintained in a 1:1 mixture of Ham’s F12 medium and Dulbecco’s modified Eagle’s medium containing 2.5 mM l-glutamine (without phenol red) supplemented with 10% FBS and 0.3 g/L G418. Cells were cultured in a humidified incubator with 95% air and 5% CO_2_ at 34 °C.

### Quantitative reverse transcription PCR (qRT-PCR)

Total RNA was extracted using TRIzol Reagent (Promega, Madison, WI, USA) following the manufacturer’s protocols. To measure miR-338-3p expression, 1 μg of total RNA was reverse-transcribed using specific stem-loop RT primers and Mir-X™ miRNA First Strand Synthesis Kit (Takara, Dalian, China). qRT-PCR was performed using Mir-X™ miRNA qRT-PCR SYBR^®^ Kit (Takara, Dalian, China). The internal control for the detection of miR-338-3p is U6. The primers for miR-338-3p and U6 were as follow: miR-338-3p forward 5′-TGCGGTCCAGCA TCAGTGAT-3′ miR-338-3p reverse 5′-CCAGTGCAGGGT CCGAGGT-3′ U6 forward 5′-GCTCGCTTCGGC AGCACA-3′ U6 reverse 5′-GAGGTATTCGCA CCAGAGGA-3′.

To determine AHSA1 mRNA levels, 1 μg of total RNA was reverse-transcribed into cDNA, using the AffinityScript QPCR cDNA Synthesis Kit (Agilent Technologies, Inc., Santa Clara, CA, USA). qRT-PCR was performed using the Brilliant II SYBR Green QPCR Master Mix Kit (Agilent Technologies, Inc.). Amplification was performed using the following PCR profile: preheating at 95 °C for 10 min, followed by 40 cycles of 95 °C for 10 s, 60 °C for 20 s, and 72 °C for 10 s. PCR reactions were performed on an ABI PRISM^®^ 7500 Sequence Detection System (Foster City, CA, USA). Gene expression was measured in triplicate, quantified by the 2^−ΔΔCt^ method [16], and normalized using GAPDH as internal control. PCR primer sequences targeting AHSA1 and GAPDH were as follows: AHSA1 forward 5′-AGAGGGACACTTTGCCACCA-3′, reverse 5′-CTCGACCTTCCATGCACAGCT-3′; GAPDH forward 5′-ACACCCACTCCTCCACCTTT-3′; GAPDH reverse 5′’-TTACTCCTTGGAGGCCATGT-3′.

### miR-338-3p mimics, transient transfection, and AHSA1 overexpression

The miR-338-3p mimics and negative control were purchased from RIBOBIO (Guangzhou, China). Cells were plated to 50% confluency and transfected with 200 nM miR-338-3p mimic or negative control (NC), using Lipofectamine 2000 (Invitrogen) following the manufacturer’s protocol. Cells were harvested for use in further experiments 24 or 48 h after transfection. The full-length AHSA1 (NM_012111.2) gene was cloned and inserted into the expression plasmid pcDNA3.0. Transfection was performed using Lipofectamine 2000 (Invitrogen, USA) following the manufacturer’s instructions.

### Cell proliferation assay

Cell proliferation was monitored using the Cell Counting Kit-8 (CCK-8; Promega) following the manufacturer’s protocol. At 24 h after transfection, MG-63 and Saos2 cells were seeded at 1 × 10^3^ per well in 96-well plates. Cell proliferation assay was performed on days 1, 2, and 3. After adding 10 μL of WST reagent to each well, the plate was incubated for 4 h at 37 °C. Before the endpoint of incubation, absorbance was measured at 450 nm using a Vmax microplate spectrophotometer (Molecular Devices, Sunnyvale, CA). Each sample was assayed thrice.

### Flow cytometry analysis

After treated with different condition, MG-63 and Saos2 cells were dissociated using trypsin, then centrifuged at 2000 rpm for 5 min. Next, cells were washed twice with PBS and centrifuged at 2000 rpm for 5 min. Annexin V-FITC/PI Apoptosis Detection Kit was used to analyze the apoptosis rate according to the manufacturer’s protocols (Keygen, Nanjing, China). Briefly, the cell pellet (~ 1–5 × 105 cells) was resuspended in 500 μL Binding Buffer. Then, 5 μL Annexin V-FITC and 5 μL PI were added to the cell suspension, which was gently mixed and incubated at room temperature, protected from light, for 15 min. Within 1 h, the cells were analyzed via NovoCyte Flow Cytometer (ACEA Biosciences, Inc., San Diego, CA, USA). Cell Cycle Detection Kits was used to analyze cell cycle distribution according to the manufacturer’s protocols (Keygen). Briefly, cells were fixed in 500 μL 70% ice-cold ethanol at 4 °C overnight. Cells were then washed twice with 500 μL PBS. Up to 100 μL RNaseA was added and cells were incubated at 37 °C for 30 min. Next, 100 μL PI was added and cells were incubated at 4 °C in the dark for 30 min. The cell cycle distribution was then analyzed via a Cytomics FC 500 (Beckman Coulter, Fullerton, CA, USA). Each experiment was repeated three times.

### Cell migration and invasion

MG-63 and Saos2 cells were transfected with miR-338-3p mimics, AHSA1 overexpression plasmid, or negative control (NC), and subsequently cultivated for 24 h. To monitor cell migration, transfected cells were harvested, and 5 × 10^4^ cells in 200 µL of 0.1% serum medium were placed in the upper chamber of an insert (pore size, 8 µm) (Becton–Dickinson Labware). The lower chamber was filled with 10% fetal bovine serum medium (600 µL). To monitor cell invasion, 5 × 10^4^ cells in 200 µL of 0.1% serum medium were placed in the upper chambers, which were pre-coated with Matrigel (BD Biosciences). After 24 h of incubation, cells were removed from the upper chamber of the filter, using a cotton swab. Cells on the underside were fixed with 4% paraformaldehyde, stained with 0.1% crystal violet in 20% ethanol, and counted in five randomly selected fields under a phase contrast microscope. Migrated cells were monitored by photographing at 200× magnification, using a LEICA microscope, in five independent fields per well. Assays were performed in triplicate.

### Western blotting analysis

Total proteins were extracted using RIPA Lysis Buffer (Beyotime Biotechnology, Shanghai, China) following the manufacturer’s protocol. Thirty micrograms of protein were separated by 10% SDS polyacrylamide gel electrophoresis and transferred onto PVDF membranes (Millipore, Billerica, MA, USA). Membranes were blocked for 1 h at 37 °C with 5% non-fat milk and subsequently incubated with anti-E-cadherin (1:500, Cell Signaling Technology, Irvine, CA, USA), anti-Vimentin (1:800, Cell Signaling Technology), anti-AHSA1 (1:1000 dilution, Abcam, Cambridge, MA, USA) and GAPDH (1:400 dilution, Santa Cruz Biotechnology, Santa Cruz, CA, USA) in 5% non-fat milk for 1 h at 37 °C. After washing with TBS containing 0.5% Tween 20 (TBST), membranes were incubated with HRP-conjugated secondary antibody at 37 °C for 40 min. After further washing with TBST, membranes were assayed via enhanced chemiluminescence (ECL) and recorded on X-ray films.

### Plasmid construction and luciferase reporter assay

To construct a luciferase reporter vector, the 3′UTRs of wild-type and mutant AHSA1 containing the putative miR-338-3p-binding sites were subcloned into the psiCHECK-2 vector. For the luciferase reporter assay, MG-63 and Saos2 cells were plated at 5 × 10^4^ cells per well in 24-well plates. The next day, psiCHECK-2 luciferase vectors containing the 3′UTR of AHSA1 and miR-338-3p mimics or negative control oligonucleotides were transfected into cells, using Lipofectamine 2000 (Invitrogen, Carlsbad, CA, USA). At 48 h after transfection, luciferase assays were performed using the dual luciferase reporter assay system (Promega).

### Statistical analysis

Statistical analysis was performed using SPSS 19.0 software package (SPSS Inc, Chicago, IL, USA). All numerical data were analyzed by Student t test. All tests performed were two-sided. Statistical significance was considered at P < 0.05.

## Results

### miR-338-3p is downregulated in osteosarcoma tissues and cell lines

**Fig. 1:**
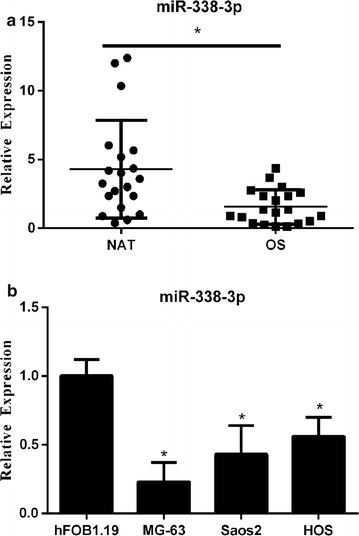
miR-338-3p is downregulated in osteosarcoma tissues and cell lines. **a** miR-338-3p is downregulated in osteosarcoma tissues. **b** miR-338-3p is downregulated in osteosarcoma cell lines. Data were expressed as mean ± SD of three independent experiments. *P < 0.05

To determine the role of miR-338-3p in osteosarcoma, qRT-qPCR was performed to quantify miR-338-3p expression levels in the tissue samples of 20 osteosarcoma patients and corresponding adjacent normal tissues (ANT) used as controls. As presented in Fig. 1a, miR-338-3p was downregulated in most osteosarcoma tissues compared with that in the corresponding ANT. Next, we determined miR-338-3p expression levels in several common osteosarcoma cell lines, namely, MG-63, Saos2, and HOS. The human osteoblast cell line hFOB1.19 was used as a control. Our results revealed that miR-338-3p was significantly downregulated in osteosarcoma cell lines when compared with that in hFOB1.19 (Fig. 1b). In addition, MG-63 and Saos2 cells showed significantly lower miR-338-3p expression levels than the hFOB1.19 cells. Therefore, MG-63 and Saos2 cells were used in subsequent experiments.

### AHSA1 is a target of miR-338-3p

**Fig. 2:**
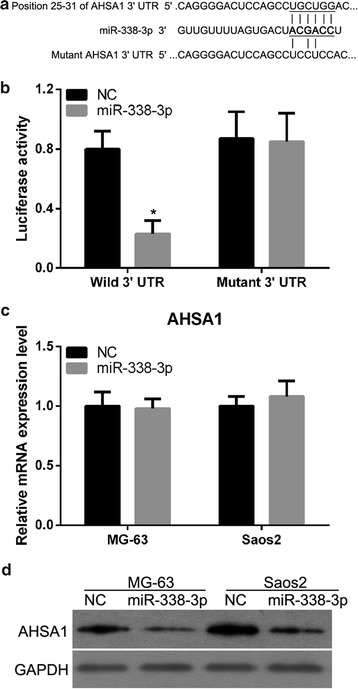
AHSA1 is a direct target of miR-338-3p. **a** Predicted duplex formation between the 3′UTRs of wild-type or mutant AHSA1 and miR-338-3p. **b** Luciferase activity of wild-type (Wild 3′UTR) or mutant (Mutant 3′UTR) AHSA1 3′UTR reporters in 293T cells transfected with a miR-338-3p mimic or NC. **c** qRT-PCR data showing AHSA1 mRNA levels in MG-63 and Saos2 cells transfected with a miR-338-3p mimic or NC. Data were normalized against GAPDH mRNA levels. **d** Western blotting of AHSA1 in MG-63 and Saos2 cells transfected with a miR-338-3p mimic or NC. Data are expressed as mean ± SD, *P < 0.05

On the basis of the identified putative conserved target sequences at positions 25–31 of the AHSA1 3′-UTR, we hypothesized that AHSA1 is a potential target of miR-338-3p (Fig. 2a). We verified whether miR-338-3p can directly bind to its seed sequences in the AHSA1 3′-UTR in 293T cells. We observed significant reduction in luciferase reporter activity in the vector containing the wild-type AHSA1 3′-UTR in the presence of miR-338-3p (Fig. 2b) when compared to that in NC. This significant decrease in reporter activity was not observed when the reporter was in the vector containing the mutant AHSA1 3′-UTR (Fig. 2b) even in the presence of miR-338-3p. These results confirm that sequences in the 25–31-bp region of the AHSA1 3′-UTR interact with miR-338-3p to inhibit AHSA1 expression. Therefore, miR-338-3p can directly bind to its seed sequence in the AHSA1 3′-UTR. We next examined the effect of miR-338-3p overexpression on mRNA and protein expression of AHSA1. miR-338-3p overexpression did not result in the degradation of AHSA1 mRNA (Fig. 2c). However, endogenous AHSA1 protein levels were evidently reduced upon miR-338-3p overexpression (Fig. 2d). Therefore, our results indicate that AHSA1 is a target of miR-338-3p in OS cells.

### miR-338-3p overexpression suppresses cell proliferation

**Fig. 3:**
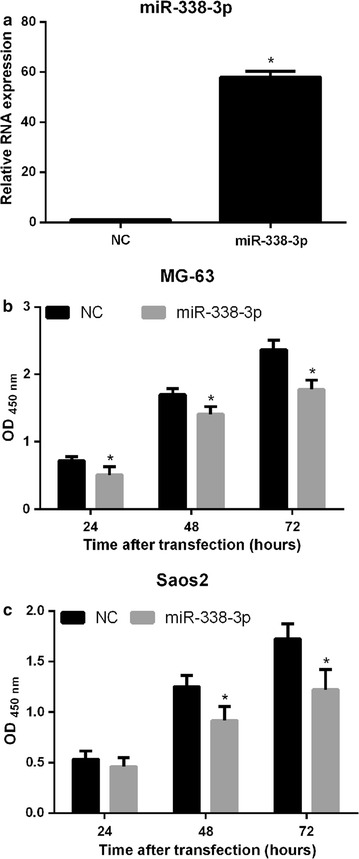
Effect of miR-338-3p overexpression on proliferation of osteosarcoma cells. MG-63 and Saos2 cells were transfected with miR-338-3p mimic or NC, and miR-338-3p expression levels were determined by quantitative reverse transcription PCR (**a**). At 24, 48, and 72 h after transfection of NC and miR-338-3p mimics, OD was determined to assess proliferation of MG-63 (**b**) and Saos2 (**c**) cells. Data are expressed as mean ± SD, *P < 0.05

MG-63 and Saos2 cells were separately transfected with miR-338-3p or NC mimics. qRT-PCR analysis was performed to determine miR-338-3p expression levels following transfection. miR-338-3p expression was found to be significantly upregulated in MG63 and Saos2 cells transfected with miR-338-3p mimics compared to that in cells transfected with NC (Fig. 3a). These results suggest that miR-338-3p was overexpressed in MG-63 and Saos2 cells.

To determine the effects of miR-338-3p overexpression on cell proliferation, CCK8 assay was performed; optical density (OD) was determined at 450 nm. Results revealed that MG-63 and Saos2 cells overexpressing miR-338-3p showed markedly reduced proliferation compared to cells transfected with NC, after 24, 48, and 72 h (Fig. 3b and c).

**Fig. 4:**
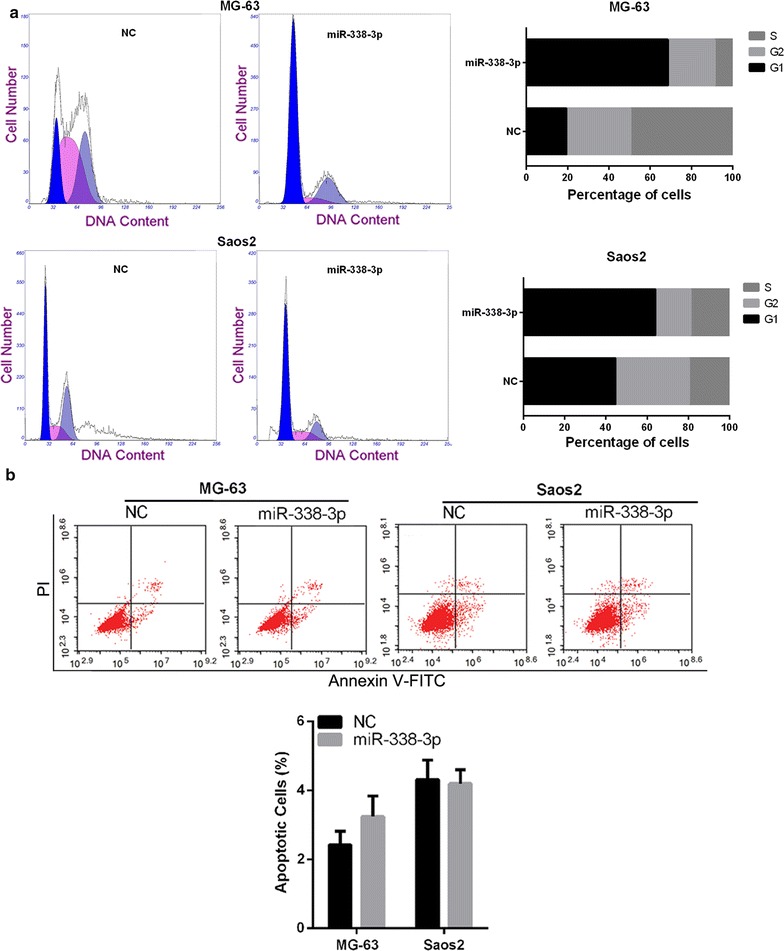
Effect of miR-338-3p overexpression on cell cycle and apoptosis of osteosarcoma cells. After transfected with miR-338-3p mimic or NC for 48 h, MG-63 and Saos2 cells were harvested for flow cytometry analysis to determine the distribution of cell cycle stages (**a**) and apoptosis (**b**)

To determine the mechanism by which miR-338-3p overexpression suppresses cell proliferation, we used flow cytometry to determine the distribution of cell cycle stages and apoptosis after miR-338-3p mimics transfection. The results showed that overexpression of miR-338-3p induced a significant G1-phase arrest in both MG-63 and Saos2 cells (Fig. 4a). In addition, we found that overexpression of miR-338-3p did not affect the apoptosis of both MG-63 and Saos2 cells (Fig. 4b).

### miR-338-3p overexpression suppresses migration, invasion, and EMT

**Fig. 5:**
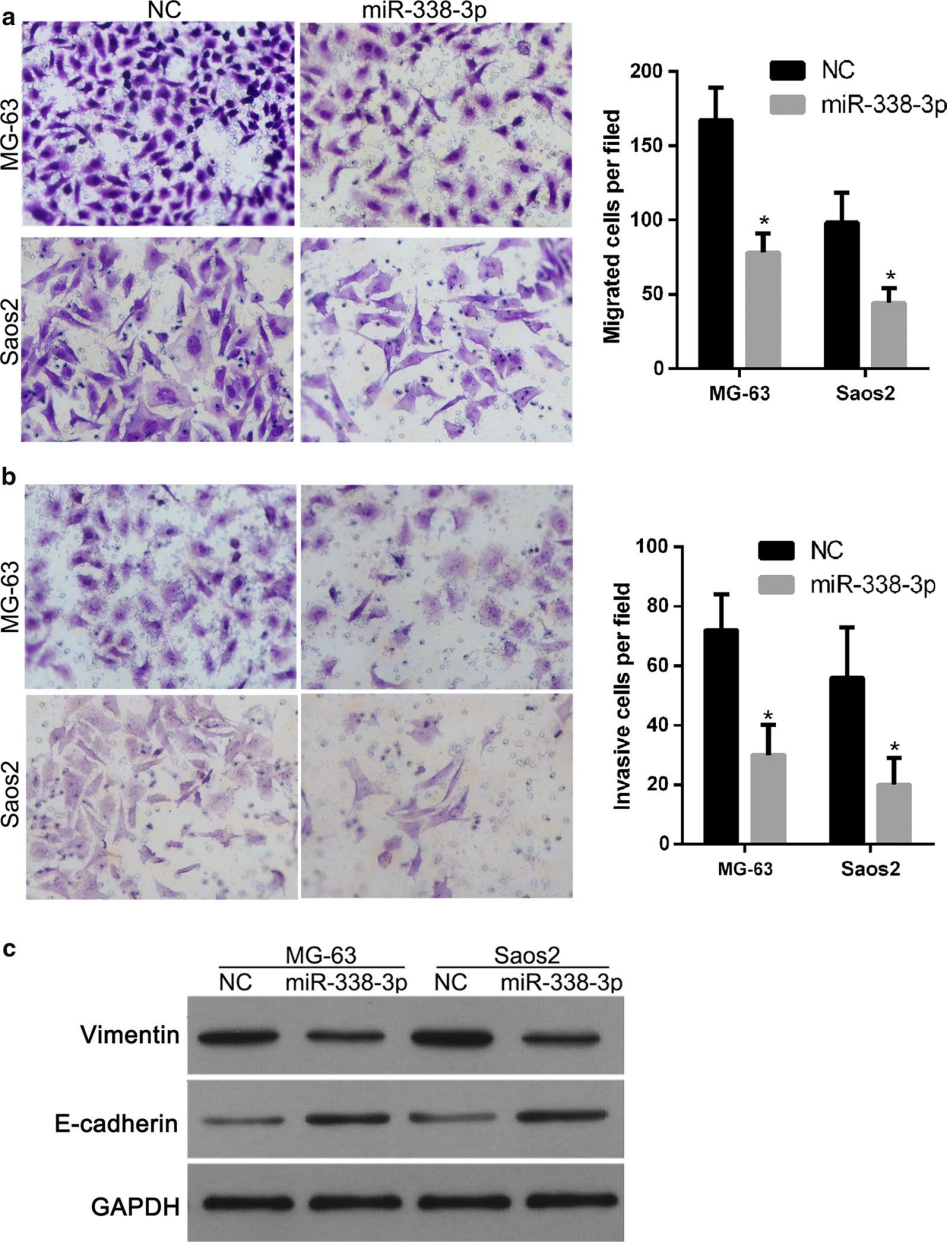
Effect of miR-338-3p overexpression on migration, invasion, and the expression of Vimentin and E-cadherin of osteosarcoma cells. Following transfection with NC and miR-338-3p mimics, transwell assay was performed to measure the migratory and invasive capacities of MG-63 (**a**) and Saos2 (**b**) cells. Left image shows representative results for migration or invasion of cells from each treatment group. Right image shows the average number of migratory or invasive cells per field among different treatment groups. Data are expressed as mean ± SD, *P < 0.05. Western blot was carried out to detect the expression of Vimentin and E-cadherin (**c**)

Transwell assay was performed to measure the migratory and invasive capacities of MG-63 and Saos2 cells. MG-63 and Saos2 cells treated with miR-338-3p mimics showed significantly lower transwell migration capacity than those treated with NC mimics (Fig. 5a). Results of the invasion assay showed that miR-338-3p overexpression significantly reduced the invasive capacity of osteosarcoma cells (Fig. 5b). These findings suggest a functional role for miR-338-3p in suppressing the migration and invasion behavior of osteosarcoma cells.

The results of western blot showed that miR-338-3p overexpression significantly increased the protein level of the epithelial marker, E-cadherin, and correspondingly decreased the levels of mesenchymal marker Vimentin (Fig. 5c). These findings suggest miR-338-3p overexpression suppresses EMT of osteosarcoma cells.

### AHSA1 mediates the effects of miR-338-3p on proliferation, migration, and invasion of MG-63 and Saos2 cells

**Fig. 6:**
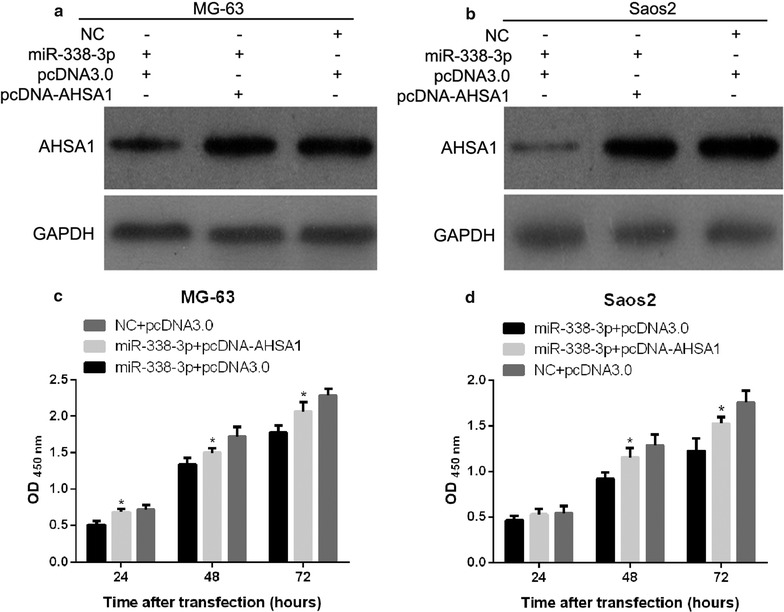
AHSA1 overexpression attenuates the effects of miR-338-3p on proliferation of osteosarcoma cells. MG-63 and Saos2 cells were transfected with miR-338-3p plus pcDNA-AHSA1, pcDNA3.0 vector plus miR-338-3p, or NC plus pcDNA3.0. AHSA1 protein expression levels were determined via western blotting (**a** and **b**). At 24, 48, and 72 h after transfection, OD was measured to assess proliferation capacities of MG-63 (**c**) and Saos2 (**d**) cells. Data are expressed as mean ± SD, *P < 0.05

In the present study, we demonstrated that AHSA1 is a target of miR-338-3p. To verify that miR-338-3p modulates cell proliferation, cell cycle, migration, and invasion of MG-63 and Saos2 by downregulating AHSA1, we determined whether AHSA1 overexpression influences the effects of miR-338-3p on proliferation, cell cycle, migration and invasion of MG-63 and Saos2 cells. We co-transfected expression plasmids containing miR-338-3p plus pcDNA-AHSA1 into MG-63 and Saos2 cells. The control group was transfected with the pcDNA3.0 vector plus miR-338-3p or NC plus pcDNA3.0. Following transfection, AHSA1 protein levels were examined via western blotting. As shown in Fig. 6a and b, cells transfected with miR-338-3p plus pcDNA-AHSA1 had higher AHSA1 protein levels than cells transfected with pcDNA3.0 vector plus miR-340-5p, and these levels were comparable to those in cells transfected with NC plus pcDNA3.0

**Fig. 7:**
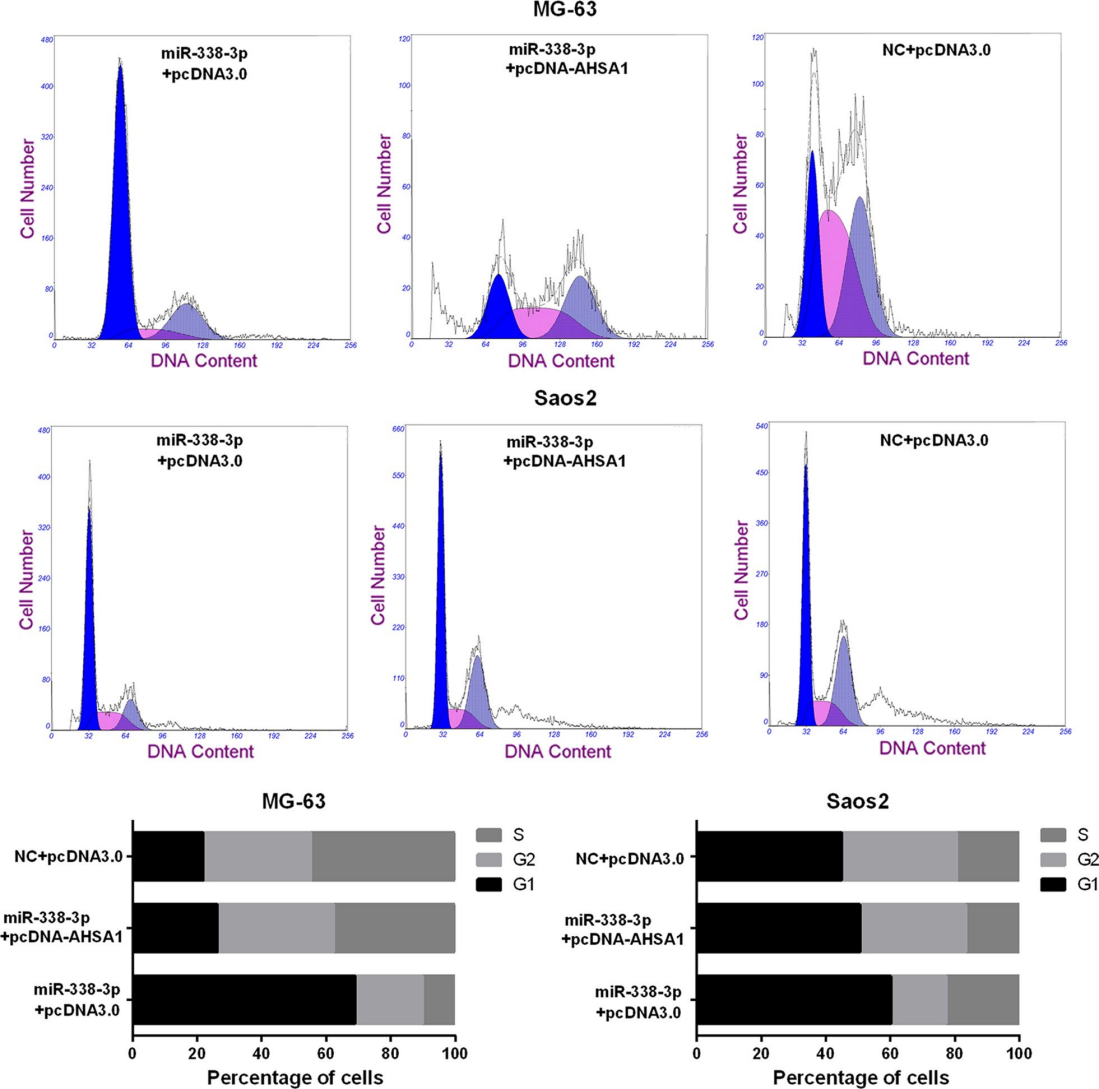
AHSA1 overexpression attenuates the effects of miR-338-3p on cell cycle of osteosarcoma cells. After transfected with miR-338-3p plus pcDNA-AHSA1, pcDNA3.0 vector plus miR-338-3p, or NC plus pcDNA3.0, MG-63 and Saos2 cells were harvested for flow cytometry analysis to determine the distribution of cell cycle stages

The obtained OD_450nm_ values of MG-63 and Saos2 cells transfected with miR-338-3p plus pcDNA-AHSA1 were found to be higher than those transfected with miR-338-3p plus pcDNA3.0 and lower than those transfected with NC plus pcDNA3.0 (Fig. 6c and d). In addition, the percentage of cells in G1-phase after transfected with miR-338-3p plus pcDNA-AHSA1 was found to be lower than those transfected with miR-338-3p plus pcDNA3.0, but is still higher than those transfected with NC plus pcDNA3.0 (Fig. 7). All results suggest AHSA1 expression attenuates the effects of miR-338-3p on proliferation of MG-63 and Saos2 cells by partial relieving G1-phase arrest.

**Fig. 8:**
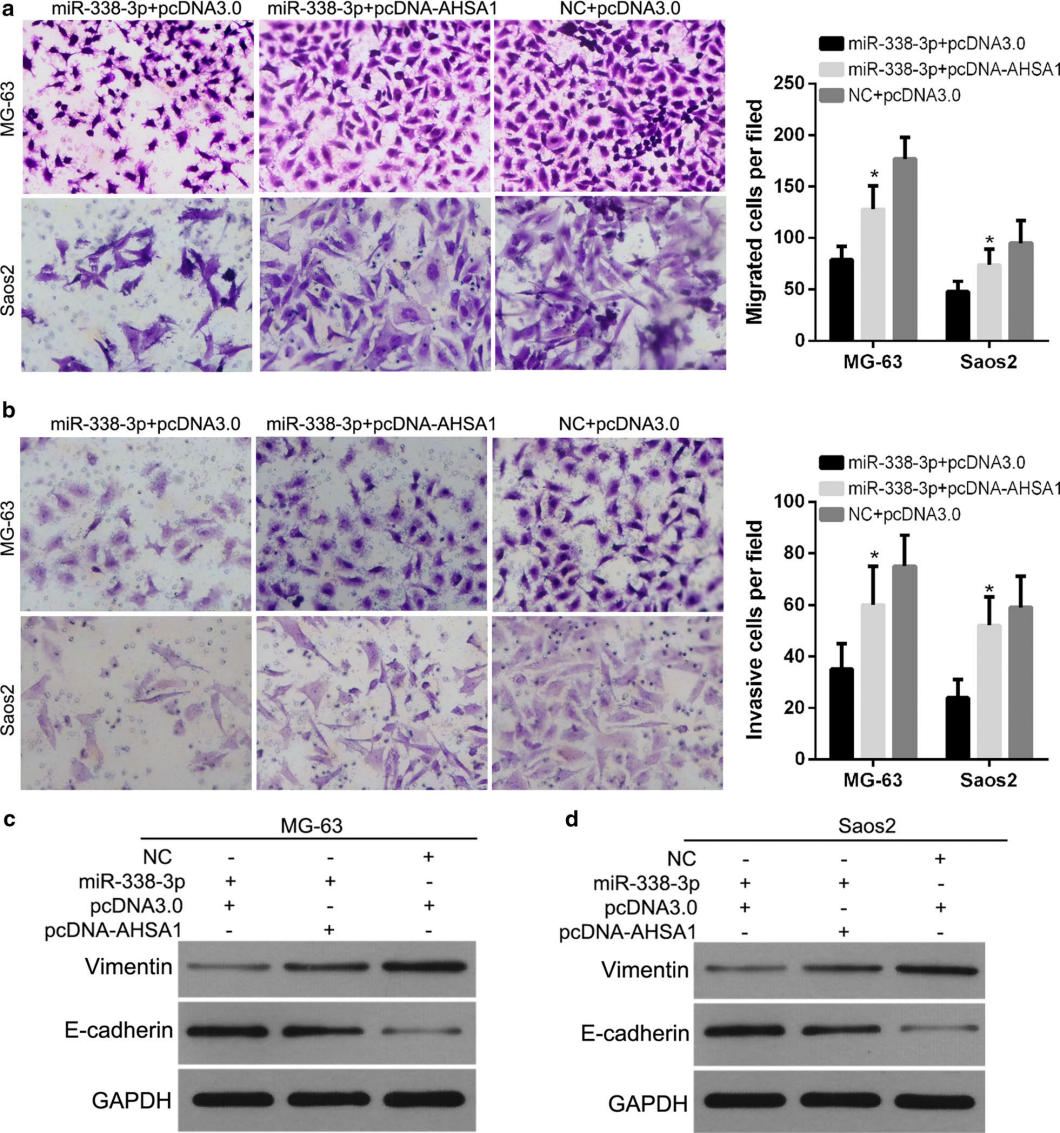
AHSA1 overexpression attenuates the effects of miR-338-3p on migratory and invasive capacities, and the expression of Vimentin and E-cadherin of osteosarcoma cells. MG-63 and Saos2 cells were transfected with miR-338-3p plus pcDNA-AHSA1, pcDNA3.0 vector plus miR-338-3p, or NC plus pcDNA3.0. Transwell assay was performed to measure the migratory and invasive capacities of MG-63 (**a**) and Saos2 (**b**) cells. Left image shows representative results of migration or invasion capacities of cells from each treatment group. Right image shows the average number of migrated or invading cells per field in each treatment group. Data are expressed as mean ± SD, *P < 0.05. Western blot was carried out to detect the expression of Vimentin and E-cadherin in MG-63 (**c**) and Saos2 (**d**) cells

We also investigated whether AHSA1 influences the inhibitory effect of miR-340-5p on the migratory and invasive capacities of MG-63 and Saos2 cells. For the transwell migration assay, the number of cells that crossed the membrane onto the lower chamber was found to be significantly higher for cells transfected with miR-338-3p plus pcDNA-AHSA1 than for those transfected with miR-338-3p plus pcDNA3.0 (Fig. 8a). Results of the transwell invasion assays showed that the number of cells that crossed the Matrigel-coated membrane onto the lower chamber were higher for cells transfected with miR-338-3p plus pcDNA-AHSA1 than for cells transfected with miR-338-3p plus pcDNA3.0 (Fig. 8b).

In addition, we found that the expression of Vimentin in MG-63 and Saos2 cells transfected with miR-338-3p plus pcDNA-AHSA1 was found to be higher than those transfected with miR-338-3p plus pcDNA3.0, but is still lower than those transfected with NC plus pcDNA3.0 (Fig. 8c). And the expression of E-cadherin in MG-63 and Saos2 cells transfected with miR-338-3p plus pcDNA-AHSA1 was found to be lower than those transfected with miR-338-3p plus pcDNA3.0, but is still higher than those transfected with NC plus pcDNA3.0 (Fig. 8c). These results indicated that AHSA1 expression could attenuate the effects of miR-338-3p on EMT of MG-63 and Saos2 cells.

## Discussion

miRNAs can serve as regulators of various critical biological processes [17]. In tumor cells, miRNAs can play multiple roles as tumor suppressors, oncogenes, or both in some cases [18]. To date, many miRNAs involved in OS have been described, suggesting that miRNA-based therapeutic strategies can serve as novel therapeutic treatments to restore or inhibit the expression of mRNAs involved in OS. However, further research is required to validate the correlations between specific miRNAs and OS. In the present study, we first studied the association between miR-338-3p and OS to explore a potential therapeutic target for OS treatment. We demonstrated that miR-338-3p is downregulated in OS patient tissues and OS cell lines. In addition, miR-338-3p overexpression was found to reduce viability, EMT, migration, and invasion of the OS cell lines MG63 and Saos2. Previous studies have demonstrated that miR-338-3p is involved in the progression of several cancers, including colorectal carcinoma, neuroblastoma, gastric cancer, non-small cell lung cancer, ovarian cancer, and hepatocellular carcinoma [12–15, 19–21]. Although miR-338-3p exerts diverse biological effects in several cancers that varies depending on cell type, miR-338-3p has been demonstrated to act as a tumor suppressor in the abovementioned cancer cell types [12–15, 19–21]. Our results not only suggest a tumor suppressive role for miR-338-3p in the development of OS, but also identify a putative gene target for therapeutic treatment of OS.

In our previous study, AHSA1 silencing was demonstrated to inhibit growth, migration, and invasion and increased apoptosis of MG-63 and Saos2 cells, thereby suggesting that AHSA1 functions as an oncogene in OS [6]. In addition, a miR-338-3p-binding site was identified in the 3′UTR of AHSA1. Together with the contradictory effects of AHSA1 and miR-338-3p overexpression, results of our study indicate that miR-338-3p might regulate AHSA1 by targeting mRNAs for cleavage or translational repression. Results of luciferase reporter assay, qRT-PCR, and western blotting indicate that miR-338-3p can suppress AHSA1 protein expression but does not significantly affect AHSA1 mRNA levels. Our results revealed that miR-338-3p can inhibit AHSA1 expression by targeting mRNAs for translational repression. We also provide evidence that AHSA1 overexpression reverses the inhibitory effects of miR-338-3p on proliferation, EMT, migration, and invasion abilities of the OS cell lines MG63 and Saos2. However, AHSA1 overexpression did not completely reverse the effects of miR-338-3p, indicating that miR-338-3p inhibits proliferation, EMT, migration, and invasion in OS cells partially by targeting AHSA1. In other cancer cells, many genes have been identified as miR-338-3p targets, including ADAM17, PREX2a, ZEB2, Sox4, SMO, MACC1, and IRS2 [12–15, 19–21]. Therefore, miR-338-3p can potentially inhibit proliferation, EMT, migration and, invasion of OS cells through other targets.

## Conclusions

In conclusion, our study provides in vitro evidence that miR-338-3p inhibits OS cell proliferation, EMT, migration, and invasion by downregulating AHSA1 expression. However, further studies using animal models are required to verify our current findings. We examined the expression patterns of miR-338-3p in a small number of OS tissue samples. A larger sample size is needed to investigate the clinical significance of miR-338-3p. We will examine the relationship between miR-338-3p and clinical pathological parameters of OS in future studies. In addition, several issues remain to be resolved, such as determining whether miR-338-3p targets other genes, elucidating the mechanisms underlying the regulatory effect of miR-338-3p on AHSA1 and other target genes in OS, and evaluating the potential of miR-338-3p as a therapeutic target in OS.

## Abbreviations

OS: osteosarcoma
qRT-PCR: quantitative reverse transcription PCR
AHSA1: activator of 90 kDa heat shock protein ATPase homolog 1
miRNAs: microRNAs
mRNA: messenger RNA
3′UTRs: 3′-untranslated regions
NSCLC: non-small cell lung cancer
NAT: normal adjacent tissues
CCK-8: Cell Counting Kit-8
OD: optical density
EMT: epithelial–mesenchymal transition
