# Correction to: microRNA-338-3p inhibits proliferation, migration, invasion, and EMT in osteosarcoma cells by targeting activator of 90 kDa heat shock protein ATPase homolog 1

**DOI:** 10.1186/s12935-021-02283-2

**Published:** 2021-10-26

**Authors:** Riliang Cao, Jianli Shao, Yabin Hu, Liang Wang, Zhizhong Li, Guodong Sun, Xiaoliang Gao

**Affiliations:** 1 Department of Pediatric Surgery, Guangdong Women and Children Hospital, Guangzhou, 511400 China; 2 Department of Orthopedic and Traumatology, First Affiliated Hospital, Jinan University, Guangzhou, 510632 China; 3 Department of Spinal Surgery, The Sixth Affiliated Hospital of Xinjiang Medical University, Ürümqi, 830002 Xinjiang China; 4 Department of Oncology, First Affiliated Hospital, Jinan University, Guangzhou, 510632 China

## Correction to: Cancer Cell Int (2018) 18:49 10.1186/s12935-018-0551-x

Following the publication of the original article [1], we were notified that the representative images for migration of Saos2 cells in Fig. 5A had been misused. The statistics are not wrong and the mistake doesn't affect the conclusion of the paper. The correct Fig. 5 and caption are shown in this correction.

**Fig. 5:**
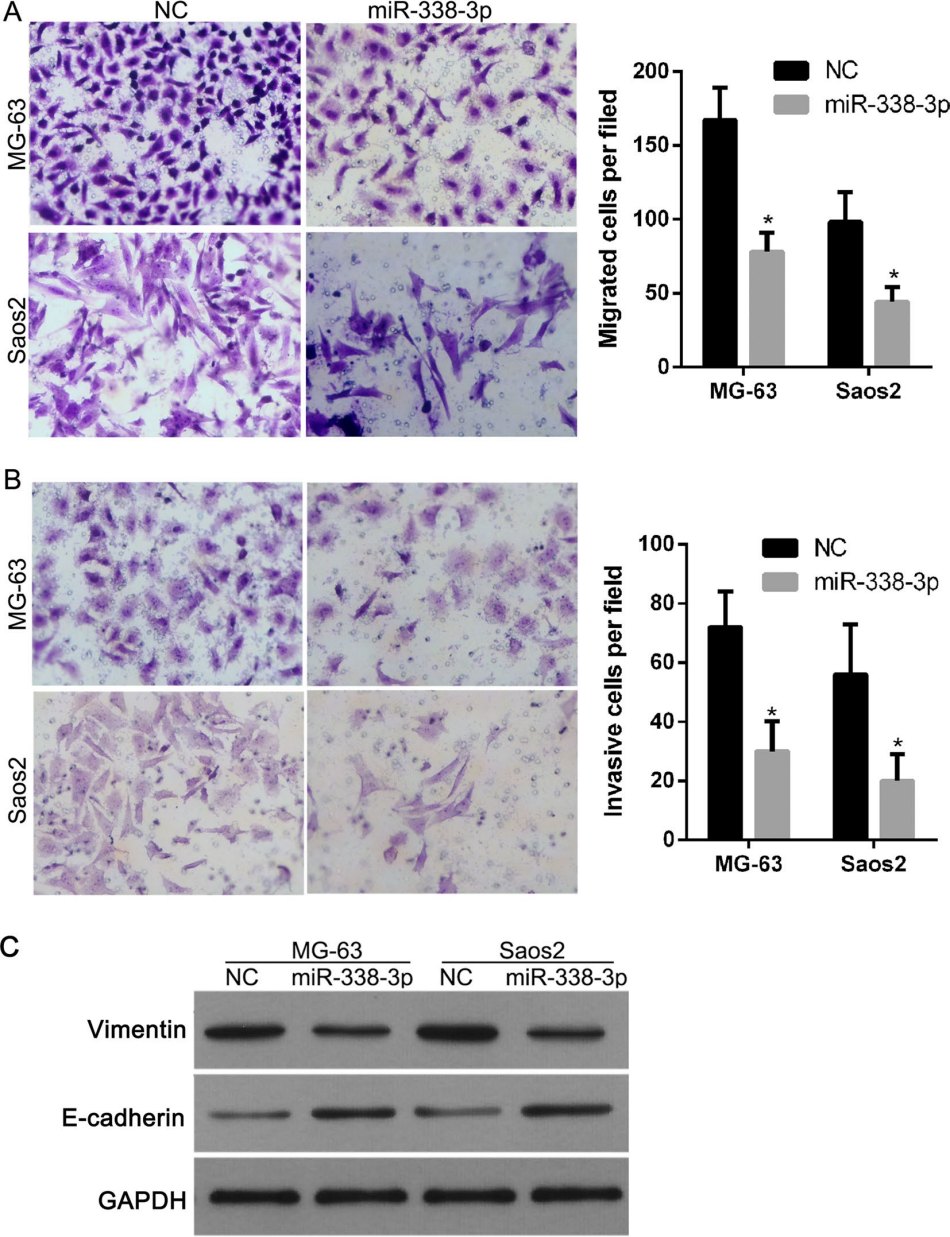
Effect of miR-338-3p overexpression on migration, invasion, and the expression of Vimentin and E-cadherin of osteosarcoma cells. Following transfection with NC and miR-338-3p mimics, transwell assay was performed to measure the migratory (**A**) and invasive (**B**) capacities of MG-63 and Saos2 cells. Left image shows representative results for migration or invasion of cells from each treatment group. Right image shows the average number of migratory or invasive cells per field among different treatment groups. Data are expressed as mean ± SD, *P < 0.05. Western blot was carried out to detect the expression of Vimentin and E-cadherin (**C**)
